# Functional Interpretation of a Novel Homozygous METTL5 Variant Associated with ADHD and Neurodevelopmental Abnormalities: A Case Report and Literature Review

**DOI:** 10.3390/genes16121502

**Published:** 2025-12-15

**Authors:** Sheema Hashem, Saba F. Elhag, Ajaz A. Bhat, Waleed Aamer, Aljazi Al-Maraghi, Hala Alhaboub, Dalya Abuthaher, Ammira S. Al-Shabeeb Akil, Mohammad Haris, Khalid Fakhro, Georges Nemer, Madeeha Kamal

**Affiliations:** 1 College of Health and Life Sciences, Hamad Bin Khalifa University (HBKU), Doha P.O. Box 5825, Qatar; shashem@sidra.org (S.H.); selmubarakelhag@sidra.org (S.F.E.); 2 Department of Human Genetics, Sidra Medicine, Doha P.O. Box 26999, Qatar; waamer@sidra.org (W.A.); aalmaraghi@sidra.org (A.A.-M.); kfakhro@sidra.org (K.F.); 3 Metabolic and Mendelian Disorders Clinical Research Program, Precision OMICs Research & Translational Science, Sidra Medicine, Doha P.O. Box 26999, Qatar; abhat@sidra.org (A.A.B.);; 4 Department of Pediatrics, Sidra Medicine, Doha P.O. Box 26999, Qatar; halasalhaboub@gmail.com (H.A.); daliaabuthaher@gmail.com (D.A.); 5 Center for Advanced Metabolic Imaging in Precision Medicine, Department of Radiology, Perelman School of Medicine, University of Pennsylvania, Philadelphia, PA 19104, USA; mohammad.haris@pennmedicine.upenn.edu; 6 Weill Cornell Medical College–Qatar (WCM-Q), Cornell University, Doha P.O. Box 24144, Qatar; 7 College of Medicine, QU Health, Qatar University, Doha P.O. Box 2713, Qatar

**Keywords:** Methyltransferase Like 5, attention-deficit/hyperactivity disorder, neurodevelopmental disorder, microcephaly, RNA methylation, whole-genome sequencing, consanguinity, trio analysis, novel variant

## Abstract

**Background and Clinical Significance:** Methyltransferase-like protein 5 (*METTL5*) is a conserved RNA methyltransferase responsible for catalyzing the N6-methyladenosine (m6A) modification of 18S ribosomal RNA, a process critical for ribosome biogenesis and translational regulation. Biallelic variants in *METTL5* have been linked to autosomal recessive intellectual developmental disorder-72 (MRT72), typically presenting with microcephaly, intellectual disability, and speech delay. However, the association between *METTL5* and isolated attention-deficit/hyperactivity disorder (ADHD) remains underexplored. **Case Presentation:** We report a 14-year-old Qatari female, born to consanguineous parents, who presented with microcephaly, speech delay, learning difficulties, and inattentive-type ADHD. Trio-based whole-genome sequencing identified a novel homozygous *METTL5* variant (c.617G > A; p. Arg206Gln), with both parent’s heterozygous carriers. The variant is extremely rare (gnomAD MAF: 0.0000175) and predicted to be deleterious (CADD: 23.7; SIFT: damaging; PolyPhen-2: probably damaging). Structural modeling localized the change within the SAM-dependent catalytic domain, predicting protein destabilization (ΔΔG = +1.8 kcal/mol). The affected residue is highly conserved (ConSurf score: 8), and protein–protein interaction analysis linked *METTL5* with *METTL14*, *METTL16*, and *ZCCHC4*, key regulators of rRNA methylation. **Conclusions:** In silico evidence suggests that the p. Arg206Gln variant disrupts *METTL5* function, likely contributing to the observed neurodevelopmental phenotype, including ADHD. This expands the clinical spectrum of *METTL5*-related disorders and supports its inclusion in neurodevelopmental gene panels.

## 1. Introduction and Clinical Significance

Intellectual disability (ID) is a clinically and genetically heterogeneous neurodevelopmental disorder affecting approximately 1–3% of the global population [1]. With the advent of next-generation sequencing technologies, the identification of rare, high-impact variants has substantially advanced our understanding of monogenic forms of ID.

**Figure 1:**
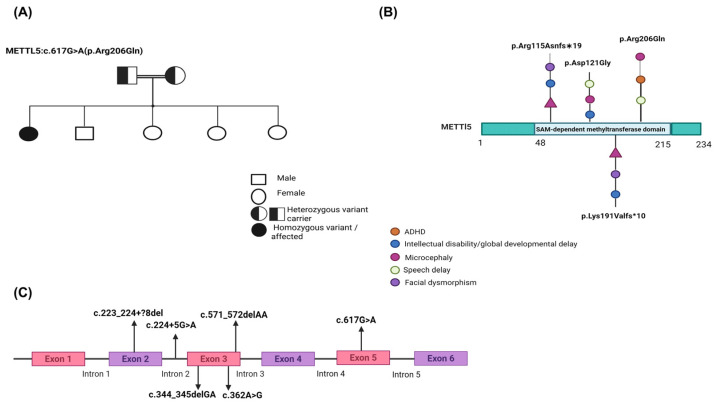
Clinical and genomic overview of the *METTL5* variant identified in this study. (**A**) Gene structure of *METTL5* with exon-intron boundaries and variant positions. The *METTL5* gene comprises six exons. Variants from previously reported cases and the current study are shown with their positions and HGVS nomenclature. The novel c.617G > A variant falls within exon 5. Other mutations shown include splice site variants (e.g., c.224 + 5G > A), frameshift deletions (e.g., c.344_345delGA, c.571_572delAA), and a missense variant (c.362A > G). (**B**) Schematic representation of the *METTL5* protein and associated pathogenic variants. Previously reported METTL5 variants are mapped along the protein, including frameshift (p.Arg115Asnfs*19*, p.Lys191Valfs10), missense (p.Asp121Gly), and splice-site variants. The novel p.Arg206Gln variant identified in our patient is located in the C-terminal region of the SAM-dependent methyltransferase domain (residues 48–215). Phenotypic features associated with each variant are represented using color-coded symbols: ADHD (orange), intellectual disability/developmental delay (blue), microcephaly (purple), speech delay (green), and facial dysmorphism (violet). (**C**) Pedigree of the affected family showing autosomal recessive inheritance. The proband (filled circle) is homozygous for the *METTL5* c.617G > A (p.Arg206Gln) variant. The parents are first cousins and both heterozygous carriers (half-filled symbols), consistent with autosomal recessive inheritance. One male sibling has a history of speech delay and mild learning difficulties.

Among the genes recently implicated in autosomal recessive syndromic ID is *METTL5* (Methyltransferase Like 5) (Figure 1A). *METTL5* encodes a conserved RNA methyltransferase responsible for catalyzing N6-methyladenosine (m6A) modification at position A1832 of 18S ribosomal RNA (rRNA), a modification essential for ribosome biogenesis and the translation of transcripts critical for neurodevelopment (Figure 1B) [2]. *METTL5* functions in concert with the cofactor TRMT112, which stabilizes the protein and enhances its methyltransferase activity. Pathogenic biallelic variants in *METTL5* have been associated with autosomal recessive intellectual developmental disorder-72 (MRT72; OMIM #618665) [3]. Affected individuals typically present with microcephaly, GDD, moderate to severe ID, speech delay, and behavioral disturbances. In some cases, dysmorphic facial features, growth abnormalities, and psychiatric symptoms have also been observed.

The initial report by Richard et al. (2019) [4] identified homozygous frameshift variants in *METTL5* among multiple consanguineous families, linking these mutations to severe ID, microcephaly, and behavioral issues such as hyperactivity and aggression. Subsequent reports have expanded the mutational and phenotypic spectrum of *METTL5*-related disorders. In China, a homozygous intronic variant (c.224 + 5G > A) was shown to cause exon 2 skipping, resulting in a 115 bp deletion that disrupted protein function and caused GDD with primary microcephaly (Figure 1A) [5]. In Afghanistan, a consanguineous family was reported with a novel homozygous missense variant (c.362A > G; p.Asp121Gly), with affected individuals exhibiting ID, microcephaly, speech delay, delayed walking, aggressive behavior, and characteristic facial features (Figure 1A) [6].

Additional cases include a Pakistani and Yemenite origin; they identified two homozygous frameshift variants in the *METTL5* gene: c.344_345delGA (p.Arg115Asnfs19) and c.571_572delAA (p.Lys191Valfs10) (Figure 1A). Both variants segregated in an autosomal recessive manner and were associated with moderate to severe intellectual disability, microcephaly, and distinct craniofacial dysmorphic features [4]. A further case from Iran described a 13-year-old boy with microcephaly and ID carrying a novel homozygous 10 bp deletion (c.223_224?8del) in the donor splice site of exon 2 of *METTL5* [7]. This variant met ACMG criteria for pathogenicity and provided additional insight into the syndrome’s variable expressivity (Figure 1A).

Collectively, these reports highlight both the genotypic diversity and phenotypic variability of *METTL5*-related neurodevelopmental disorders. Notably, several cases have reported behavioral symptoms overlapping with attention-deficit/hyperactivity disorder (ADHD), suggesting that *METTL5* may play a role in broader neuropsychiatric phenotypes. ADHD is a common, multifactorial neurodevelopmental disorder characterized by inattention, hyperactivity, and impulsiveness. While environmental factors are contributory, rare monogenic variants such as those in *METTL5* may offer novel insights into the genetic architecture of ADHD.

Here, we present the first known case of a child with formally diagnosed ADHD harboring a homozygous *METTL5* missense variant (c.617G > A; p. Arg206Gln). We combine detailed clinical phenotyping with trio-based whole-genome sequencing and a multi-pronged in silico approach to provide functional insight into the variant’s likely pathogenic role.

## 2. Case Presentation

The proband is a 14-year-old Qatari female born to healthy consanguineous parents (first cousins) (Figure 1C). The pregnancy was unremarkable, with term delivery and normal birth parameters. Postnatal assessments revealed persistent microcephaly (head circumference <3rd percentile), and early developmental milestones were delayed. Sitting occurred at 10 months, walking at nearly 2 years, and speech development was significantly delayed. By school age, the patient demonstrated notable attention difficulties, distractibility, and impaired academic performance. Hyperactivity and impulsivity were absent. At age 6, she was formally diagnosed with ADHD, predominantly inattentive type, and initially treated with methylphenidate with partial benefit. Due to side effects, treatment was intermittently paused. Neurological evaluation showed mild hypotonia and impaired fine motor coordination. Audiology and ophthalmologic evaluations were unremarkable. She remains highly dependent on daily activities. Physical examination revealed subtle dysmorphic features, including a broad nasal bridge and posteriorly rotated ears. Her stature was below the 5th percentile, and she displayed no evidence of seizures or sensory impairment. The family history revealed a male sibling with mild ID and delayed speech, but both parents were unaffected. Trio-based WGS was pursued after initial genetic testing revealed a 9p24.2 duplication of uncertain significance.

## 3. Methods and Results

### 3.1. DNA Extraction and Sequencing

This study was approved by the Sidra Medicine IRB (Number 1736896). Peripheral blood samples were collected from the proband and her parents after written informed consent was obtained, in accordance with institutional ethics guidelines. Approximately 5 mL of whole blood was drawn from each individual into EDTA tubes to prevent coagulation and maintain DNA integrity. The samples were immediately transferred to the Omics Core Facility at Sidra Medicine research department, where genomic DNA was extracted using standard protocols. WGS was performed on the NovaSeq platform, generating high-quality paired-end reads. Alignment to the GRCh38 reference genome and variant calling were completed using BWA-MEM and GATK pipelines. Variants were filtered based on quality, frequency (gnomAD < 1%), inheritance (homozygous), and relevance to neurodevelopmental phenotypes (Supplementary Figure S1A–C).

### 3.2. Variant Annotation

The homozygous missense variant c.617G > A (p.Arg206Gln) in METTL5 was identified. This variant is absent from ClinVar and extremely rare in gnomAD (MAF 0.0000175). Pathogenicity was assessed using CADD (score: 23.7), PolyPhen-2 (probably damaging), SIFT (damaging), and PROVEAN (deleterious) (Table 1).

### 3.3. Structural and Domain Analysis

**Figure 2:**
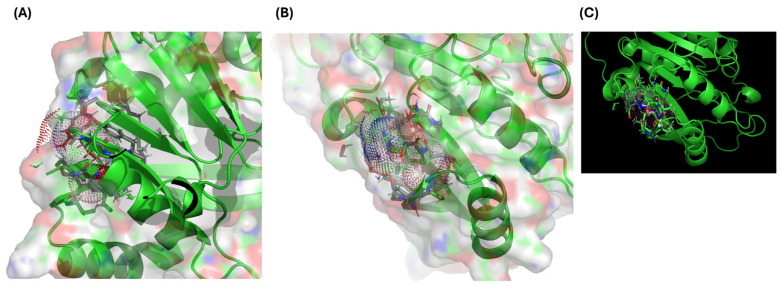
Comparative structural modeling of wild-type and p.Arg206Gln METTL5 proteins generated using AlphaFold and visualized in PyMOL (version 2.3). (**A**,**B**) show the SAM-binding pocket with Arg206 forming hydrogen bonds with the cofactor. (**C**) shows that the mutant model exhibits a loss of electrostatic interactions and the emergence of steric clashes, leading to a predicted destabilization (ΔΔG = +1.8 kcal/mol).

InterProScan identified the SAM-dependent methyltransferase domain within which Arg206 resides. Using AlphaFold (AF-Q9NRN9-F1-modelv4.pdb), residue 206 was mapped to the predicted catalytic pocket. DynaMut analysis revealed destabilization (ΔΔG = +1.8 kcal/mol), characterized by the loss of hydrogen bonding and alterations in surface electrostatics (Figure 2A–C).

### 3.4. Evolutionary Conservation

ConSurf analysis (using UniRef90 and Bayesian inference) revealed a high conservation score of 8 at Arg206. PhyloP also indicated strong conservation (score 5.1). Nearby residues 203, 206, and 209 were all found to be functionally important and exposed (Supplementary Figure S2).

### 3.5. Subcellular Localization and Disorder Prediction

DeepLoc predicted nuclear localization (0.718 probability) consistent with *METTL5*’s function. WoLF PSORT corroborated nuclear targeting. IUPred3 disorder analysis showed flexibility in the C-terminal region encompassing residue 206 (score ~0.7), suggesting potential regulatory roles (Supplementary Figure S3).

### 3.6. Post-Translational Modification Analysis

NetPhos-3.1b predicted multiple high-confidence phosphorylation sites near Arg206: S197 (score 0.829), S208 (0.674), and S160 (0.997). These may influence protein conformation or interaction dynamics (Supplementary Figure S4).

### 3.7. Protein Interaction Network

**Figure 3:**
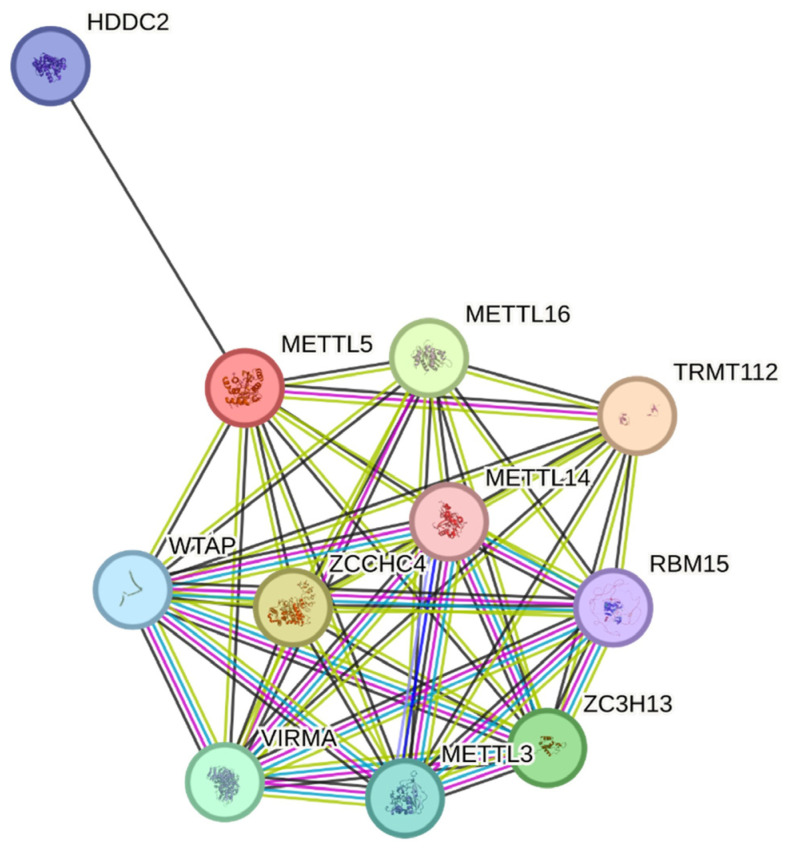
STRING protein–protein interaction network highlighting METTL5 associations with methyltransferase complex members (METTL14, METTL16, ZCCHC4, ZC3H13, TRMT112) and rRNA-modification regulators (WTAP, VIRMA, RBM15). Edge thickness represents interaction confidence (>0.9).

STRING database analysis revealed high-confidence interactions between *METTL5* and METTL16 (score 0.951), *ZCCHC4* (0.969), *METTL14* (0.721), *ZC3H13* (0.924), and *HDDC2* (0.792), consistent with its role in rRNA modification complexes (Figure 3). The network reinforces *METTL5′*s role within the epitranscriptomic methylation machinery and links functional perturbation of p.Arg206Gln to altered rRNA processing and neurodevelopmental phenotypes.

## 4. Discussion

Intellectual disability (ID) is a genetically heterogeneous disorder affecting approximately 1–3% of the global population. Advances in next-generation sequencing have identified monogenic causes such as *METTL5*, which encodes an RNA methyltransferase that modifies 18S rRNA. *METTL5* functions in conjunction with *TRMT112* and is vital for ribosome biogenesis. Biallelic pathogenic variants in *METTL5* have been associated with MRT72 (OMIM #618665), a condition characterized by microcephaly, GDD, ID, and behavioral disturbances. Several global cases have described variants ranging from missense to frameshift and splicing mutations, supporting its diverse mutational landscape and phenotypic variability (Table 2). Notably, some cases have exhibited ADHD-like traits, suggesting a broader neuropsychiatric relevance. Our report reinforces these findings by presenting a child with formally diagnosed ADHD and a homozygous *METTL5* variant.

This case describes, for the first time, a homozygous *METTL5* p.Arg206Gln variant in a child presenting with ADHD in addition to microcephaly, intellectual disability, and speech delay. Biallelic *METTL5* variants have previously been linked to autosomal recessive intellectual developmental disorder-72 (MRT72), typically characterized by microcephaly, global developmental delay, and speech impairment [4,7]. Our findings, therefore, extend the clinical spectrum of *METTL5*-related disease to include a formally diagnosed neurobehavioral phenotype.

*METTL5* encodes an m6A methyltransferase that modifies 18S rRNA at adenosine 1832, a modification essential for ribosome biogenesis and translational fidelity [2]. Loss or disruption of *METTL5* function has been shown to reduce methylation at this site, impairing ribosomal activity and altering the translation of neurodevelopmental regulators [8,9,10]. While most prior reports describe intellectual disability as the core phenotype, our case suggests that partial loss-of-function missense variants may also manifest as attentional deficits or ADHD, consistent with emerging evidence that genes encoding RNA-modifying enzymes contribute to neuropsychiatric disorders [11,12,13].

Multiple in silico analyses support the pathogenicity of the p.Arg206Gln variant. The affected arginine resides within the SAM-dependent catalytic domain of *METTL5*, and AlphaFold-based structural modeling [14] with DynaMut [15] predicts destabilization (ΔΔG = +1.8 kcal/mol), disruption of hydrogen bonds, and altered electrostatics. ConSurf [16] and PhyloP demonstrate high conservation at Arg206, indicating functional importance. NetPhos and IUPred3 highlight potential regulatory effects through phosphorylation sites (S197, S208) and increased intrinsic disorder, while STRING reveals high-confidence interactions with other epitranscriptomic regulators such as *METTL14*, *METTL16*, and *ZCCHC4* [17]. The variant is extremely rare (gnomAD MAF: 0.0000175) and absent in the homozygous state, fulfilling ACMG PM2 criteria for rarity [18].

Clinically, ADHD in the proband aligns with emerging research implicating RNA-modifying enzymes in neuropsychiatric disorders. Rare variants in epitranscriptomic regulators have been linked to ADHD and related traits, suggesting that disruptions in translational control may play a role in attentional and executive dysfunctions [9].Our findings support this idea by providing genetic and computational evidence that connects *METTL5* disruption to ADHD, indicating a broader neurobehavioral role than previously recognized.

The detection of this homozygous variant in a consanguineous Qatari family reflects population-specific patterns that increase the likelihood of identifying rare recessive alleles. Consanguinity remains an essential factor in the discovery of novel recessive variants. Qatar and other Middle Eastern countries have high rates of consanguineous marriage, increasing the likelihood of homozygous rare alleles in offspring [19,20,21,22]. This highlights the importance of trio-based genome sequencing in these populations for early diagnosis, informed management, and effective genetic counseling. In our case, recurrence risk (25%) and reproductive options were discussed with the family.

Overall, this case broadens the phenotype associated with *METTL5*-related disorders and introduces ADHD as a notable manifestation. The convergence of genetic, structural, and evolutionary evidence supports the pathogenicity of the p.Arg206Gln variant. Functional studies will be essential to confirm the mechanistic impact of this substitution on rRNA methylation and neurodevelopment.

**Table 2:** Reported Cases of *METTL5* Variants in Neurodevelopmental Disorders.

Report	Individual/Case	Variant (HGVS)	Zygosity	Inheritance	Clinical Features	Reference
Current case: Sidra Medicine	Individual	c.617G > A (p.Arg206Gln)	Homozygous	Autosomal Recessive	ADHD, speech delay, learning challenges, microcephaly and family history of learning difficulties	
	Iranian Family (3 siblings)	c.223_224 + ?8del (splice-site exon 2)	Homozygous Deletion	Autosomal Recessive	Primary microcephaly, ID, ADHD, speech delay, short stature, GDD	[7]
	Consanguineous Pakistani family	c.344_345delGA (p.Arg115Asnfs∗19)	Homozygous Frameshift	Autosomal Recessive	Moderate–severe ID, microcephaly, facial dysmorphism (e.g., large ears, dental anomalies)	[4]
	Consanguineous Yemenite family	c.571_572delAA (p.Lys191Valfs^∗^10)	Homozygous Frameshift	Autosomal Recessive	Moderate–severe ID, microcephaly, facial dysmorphism (large nose)	[4]
	Chinese family	c.224 + 5G > A (splice donor site, intron 2)	Homozygous	Autosomal Recessive	Microcephaly-related GDD	[5]
	Afghan Family (3 siblings)	c.362A > G (p.Asp121Gly)	Homozygous	Autosomal Recessive	Intellectual disability, microcephaly, poor/absent speech, delayed walking, aggressive behavior, large/posteriorly rotated ears, broad nasal base and short stature aggression, short stature, self-mutilation	[6]

## 5. Conclusions

In summary, this case broadens the phenotypic spectrum of *METTL5*-related disorders to include ADHD and provides robust bioinformatic evidence for the pathogenicity of the p.Arg206Gln variant. These findings support the inclusion of *METTL5* in neurodevelopmental gene panels and warrant functional studies to validate its mechanistic role in translational regulation and behavior.

## Figures and Tables

**Table 1:** Variant Annotation Results: METTL5 c.617G > A (p. Arg206Gln).

Genomic Position	chr14:16661234 (GRCh38)
**Annotation** **Method**	CADD v1.6
**Database**	Genome Aggregation Database (gnomAD)
**Variant Classification**	Missense (Arginine → Glutamine)
**Key Findings**	
**CADD Phred Score**	23.7 (deleterious)
**Percentile Rank**	>99% (top 1% of deleterious variants)
**Functional Impact**	Non-conservative amino acid change in the catalytic domain
Structural Implications	
**Effect 1**	Disrupts hydrogen bonding with the SAM cofactor
**Effect 2**	Loss of positive charge at the substrate recognition site
**Effect 3**	Predicted stability change (ΔΔG = +1.8 kcal/mol)
Conclusion This variant is classified as likely pathogenic based on the following evidence:	
**Criterion 1**	High CADD score (23.7 > 20 deleterious threshold)
**Criterion 2**	Location in evolutionarily conserved residue (PhyloP = 5.1)
**Criterion 3**	Critical position in the methyltransferase domain

## Data Availability

All data associated with this study are present in the paper. The datasets analyzed for patients with METTL5 mutations in this study are available through the Genome Sequence Archive at Sidra Medicine, Qatar. Due to local privacy regulations and the sensitive nature of human genomic data, access to whole-genome sequencing (WGS) data requires prior approval from the local Institutional Review Board (IRB).

## Supplementary Materials

The following supporting information can be downloaded at https://www.mdpi.com/article/10.3390/genes16121502/s1, Figure S1: IGV-based visualization of the METTL5 c.617G>A (p.Arg206Gln) variant in the proband and parents. (A) IGV screenshot of the proband (GT_66A) showing a homozygous G>A transition at chr14:170,668,340 (GRCh37/hg19), corresponding to the METTL5 c.617G>A variant. All sequencing reads display the variant allele (T), confirming homozygosity. (B) IGV view of the proband’s father (GT_66M), showing a heterozygous state for the same variant. The mix of reference (G; blue) and alternate (A; red) nucleotides across sequencing reads supports carrier status. (C) IGV image from the proband’s mother (GT_66F) also confirms heterozygous carrier status, consistent with autosomal recessive inheritance. Roughly equal representation of both alleles (G and A) is observed in the aligned reads; Figure S2: ConSurf analysis of METTL5, highlighting evolutionary conservation at residues 201–208, with Arg206 (dark purple) showing a conservation score of 8 (highly conserved). Color coding represents functional and structural importance across orthologs; Figure S3: DeepLoc-2.0 prediction confirming predominant nuclear localization (probability = 0.718) of METTL5, consistent with its role in 18S rRNA methylation. The probability threshold table shows METTL5 exceeding the nuclear cut-off (0.5014); Figure S4: Intrinsic disorder prediction and conservation profile of METTL5 obtained using IUPred3. The C-terminal region (residues 190–210) encompassing Arg206 shows increased disorder tendency (score ≈ 0.7 > 0.5 threshold).

## Abbreviations

The following abbreviations are used in this manuscript:

WGS	Whole-genome sequencing
METTL5	Methyltransferase-like 5
ADHD	Attention-deficit/hyperactivity disorder
