# Supplementary figures and images for: Functional Interpretation of a Novel Homozygous METTL5 Variant Associated with ADHD and Neurodevelopmental Abnormalities: A Case Report and Literature Review
Source: Genes (Basel). 2025 Dec 15;16(12):1502. doi: 10.3390/genes16121502 (PMC12733326; doi:10.3390/genes16121502)

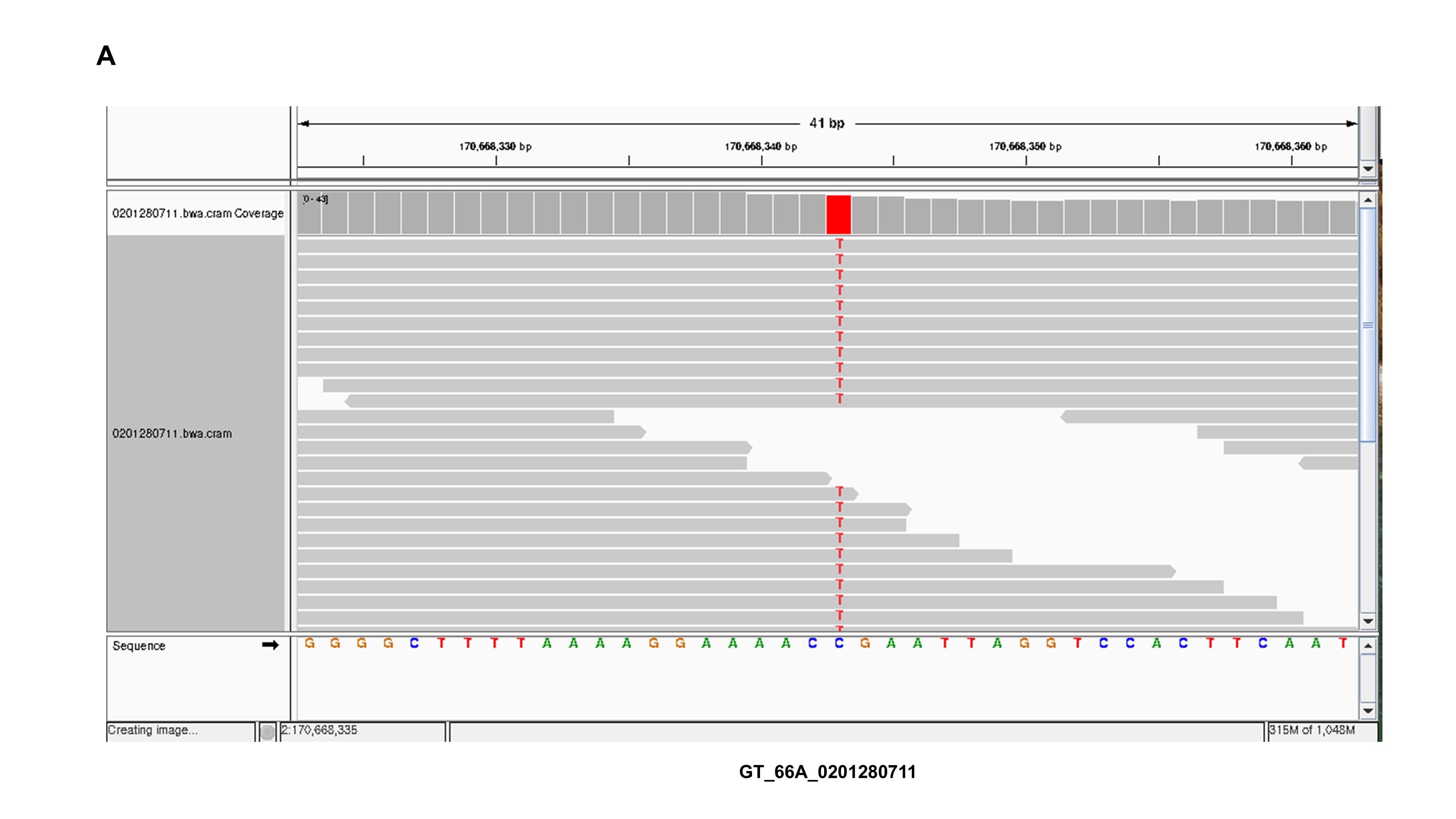

Supplement: Supplementary file 1 [file genes-16-01502-s001.zip › Supplemenatry Figure S1A.jpeg]

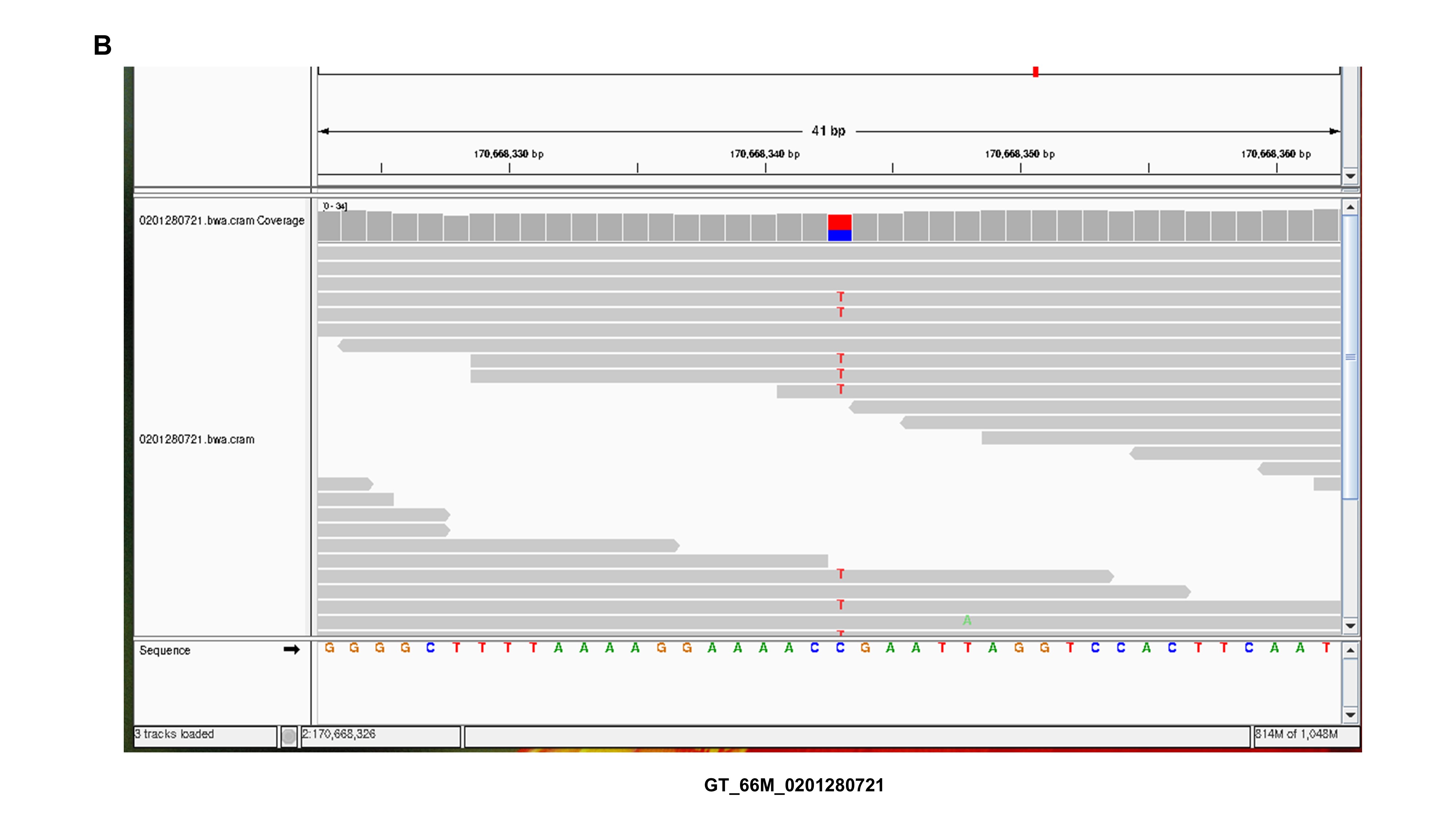

Supplement: Supplementary file 1 [file genes-16-01502-s001.zip › Supplementary Figure S1B.jpeg]

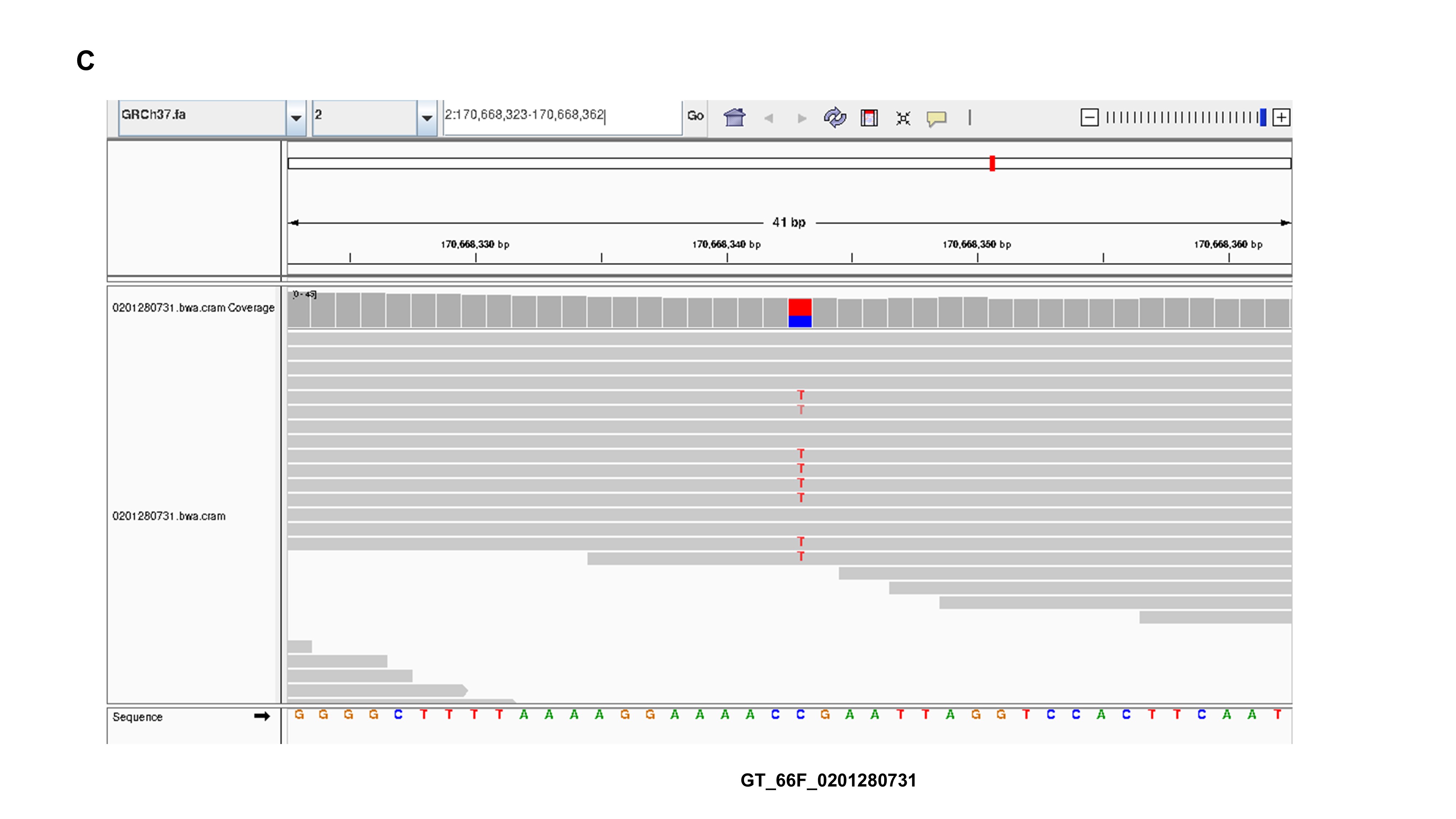

Supplement: Supplementary file 1 [file genes-16-01502-s001.zip › Supplementary Figure S1C.jpeg]

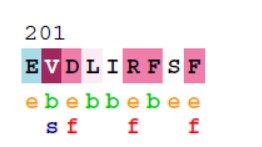

Supplement: Supplementary file 1 [file genes-16-01502-s001.zip › Supplementary Figure S2.jpeg]

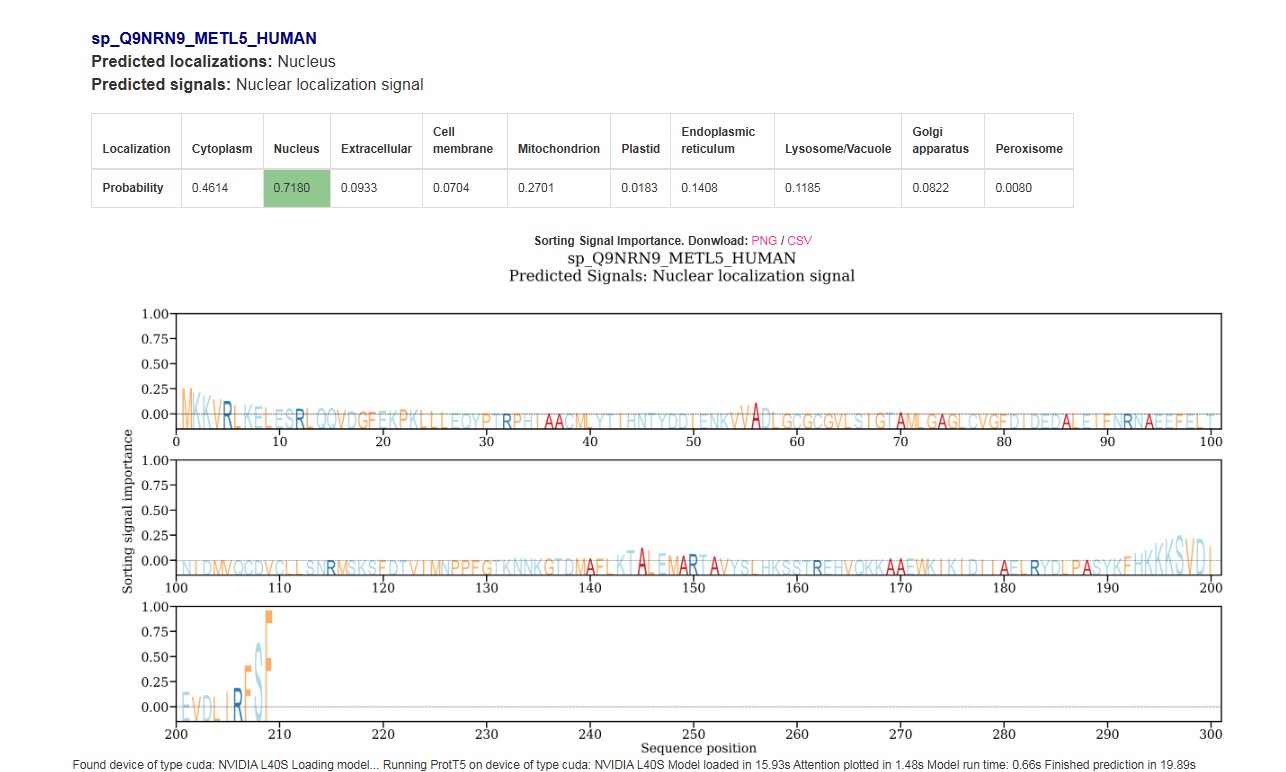

Supplement: Supplementary file 1 [file genes-16-01502-s001.zip › Supplementary Figure S3.jpeg]

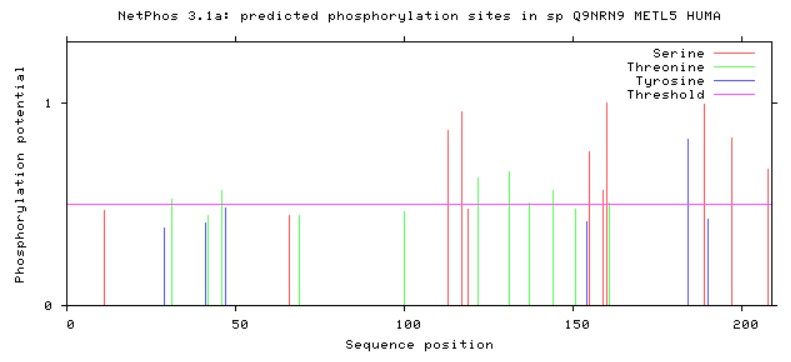

Supplement: Supplementary file 1 [file genes-16-01502-s001.zip › Supplementary Figure S4.jpeg]
